# Sub-Bandgap Sensitization of Perovskite Semiconductors via Colloidal Quantum Dots Incorporation

**DOI:** 10.3390/nano13172447

**Published:** 2023-08-29

**Authors:** G. Ribeiro, G. Ferreira, U. D. Menda, M. Alexandre, M. J. Brites, M. A. Barreiros, S. Jana, H. Águas, R. Martins, P. A. Fernandes, P. Salomé, M. J. Mendes

**Affiliations:** 1 i3N/CENIMAT, Department of Materials Science, NOVA School of Science and Technology and CEMOP/UNINOVA, Campus de Caparica, 2829-516 Caparica, Portugalm.alexandre@campus.fct.unl.pt (M.A.); s.jana@fct.unl.pt (S.J.); hma@fct.unl.pt (H.Á.);; 2 INL, International Iberian Nanotechnology Laboratory, 4715-330 Braga, Portugal; paulo.fernandes@inl.int (P.A.F.); pedro.salome@inl.int (P.S.); 3 LNEG, Estrada do Paço do Lumiar, 22, 1649-038 Lisboa, Portugal; mjoao.brites@lneg.pt (M.J.B.);; 4 CIETI, Departamento de Física, Instituto Superior de Engenharia do Porto, Instituto Politécnico do Porto, 4249-015 Porto, Portugal; 5 Departamento de Física, Universidade de Aveiro, Campus Universitário de Santiago, 3810-193 Aveiro, Portugal; 6 i3N, Universidade de Aveiro, Campus Universitário de Santiago, 3810-193 Aveiro, Portugal

**Keywords:** photovoltaics, intermediate band solar cells, Dost-in-Host hetero-crystals, perovskite semiconductors, colloidal quantum dots

## Abstract

By taking advantage of the outstanding intrinsic optoelectronic properties of perovskite-based photovoltaic materials, together with the strong near-infrared (NIR) absorption and electronic confinement in PbS quantum dots (QDs), sub-bandgap photocurrent generation is possible, opening the way for solar cell efficiencies surpassing the classical limits. The present study shows an effective methodology for the inclusion of high densities of colloidal PbS QDs in a MAPbI_3_ (methylammonium lead iodide) perovskite matrix as a means to enhance the spectral window of photon absorption of the perovskite host film and allow photocurrent production below its bandgap. The QDs were introduced in the perovskite matrix in different sizes and concentrations to study the formation of quantum-confined levels within the host bandgap and the potential formation of a delocalized intermediate mini-band (IB). Pronounced sub-bandgap (in NIR) absorption was optically confirmed with the introduction of QDs in the perovskite. The consequent photocurrent generation was demonstrated via photoconductivity measurements, which indicated IB establishment in the films. Despite verifying the reduced crystallinity of the MAPbI_3_ matrix with a higher concentration and size of the embedded QDs, the nanostructured films showed pronounced enhancement (above 10-fold) in NIR absorption and consequent photocurrent generation at photon energies below the perovskite bandgap.

## 1. Introduction

Increasing the sunlight-to-electricity conversion efficiency of solar cells is a growing technological need in view of taking the most profit from clean and free solar energy. Despite the countless strategies that have been developed in the past decades driven by such a goal, the present photovoltaic (PV) efficiencies are still far below the maximum theoretical possibilities. Therefore, new concepts need to be demonstrated and put into practice, such as the development of quantum structuring in wide-bandgap perovskite semiconductors to realize intermediate-band solar cells that enable up to 50% theoretical efficiency with a single junction [1,2,3,4,5].

Perovskite solar cells (PSCs) have been the focus of continuous research in recent years. This is mainly due to the perovskite materials’ optoelectronic properties like high absorption coefficients, high mobility, and long carrier diffusion lengths. When these optoelectronic properties are joined together with solution processability (leading to ease of fabrication), perovskite (PVK) materials become highly attractive for their use as photovoltaic absorbers [1,6,7,8,9,10,11,12,13,14]. To this day, single-junction PSCs have reached a certified maximum efficiency of 26.1% [15], which is close to the present silicon solar cells’ efficiency record of 26.8% [16]. Nevertheless, research is underway to develop different strategies to further improve photovoltaic performance. These include the engineering of perovskite inks for better stability of the deposited perovskite films [17] or the use of tandem architectures, which have already demonstrated efficiencies (e.g., 33.7% record for perovskite-on-silicon double-junctions) [16] reaching the theoretical Shockley–Queisser limit (~33%) of single-junction PV. Additionally, novel schemes like the incorporation of lower-dimensional (2D) perovskite layers are being investigated to improve interfacial properties [18]. 

**Figure 1:**
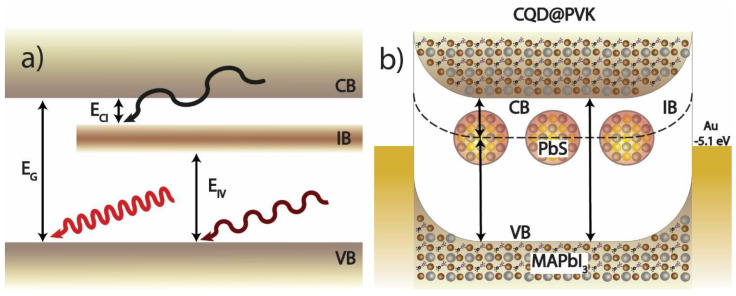
(**a**) Schematic of band transitions in an intermediate band (IB) semiconductor energy diagram, in which the IB is formed by the ground-state energy levels of the PbS CQDs embedded in a MAPbI_3_ host, as produced in this work with predicted bandgaps of: E_G_~1.6 eV, E_IV_~1.2 eV, and E_CI_~0.3 eV. (**b**) Estimated band diagram of the test devices consisting of photodetectors made of CQD@PVK IB absorbers, contacted by gold (Au) electrodes. The bandgaps and work functions (5.1 eV for Au and 4.65 eV for MAPbI_3_) represented in these sketches were informed from previous XPS analysis [40,41,42].

An alternative approach towards efficiencies above the Shockley–Queisser limit consists of the intermediate band solar cell (IBSC) concept, consisting of the introduction of an intermediate mini-band (IB) between the conduction and valence bands of the host semiconductor [19,20]. The IB enables the absorption of photons with energy below the host bandgap and the consequent generation of electron-hole pairs. This allows for a two-step photon absorption process consisting of a first electronic transition from the valence band (VB) to the IB followed by a second transition from the IB to the conduction band (CB) [1,19,20], alongside typical VB to CB photon absorption, as depicted in Figure 1a. With this mechanism, it is possible to increase the efficiency of single-junction solar cells towards a ~50% theoretical maximum at one Sun, surpassing the Shockley–Queisser limit with a single absorber [7]. This is achieved by using the extra light from the lower energy (sub-bandgap) spectrum region, mainly in the near-infrared range. 

For the practical formation of the intended IB in the active semiconductor of a photovoltaic cell, several options are being developed [4]. These include the introduction of deep-level defects in a semiconductor bulk [21,22], the split of the conduction and valence bands in highly mismatched alloys [23,24], singlet and triplet states in organic molecules [25,26], and the monodispersion of quantum dots (low-bandgap semiconductor nanoparticles) in a higher-bandgap semiconductor bulk [27,28,29], which has been the most researched approach and the one that is pursued in the present work.

Quantum dots are semiconductor nanocrystals that are capable of electronic quantum confinement [30], which is favored by high dielectric constants and large Bohr radii of the excitons [31] in the dots’ materials, as well as the low QD size [32]. Consequently, these materials are known for their discrete energy level structure, similar to a finite square well, in contrast with the continuous band structure of bulk semiconductors [30,33,34]. The ease of tuning the QDs’ absorption and emission spectra via size and composition variation has raised lots of interest for their application in a panoply of optoelectronic devices [7]. Additionally, this tunability has sparked exploration into non-conventional PV concepts such as IBSCs and multiple exciton generation (generation of multiple electron-hole pairs with the absorption of one photon) [25,35,36].

In particular, the introduction of chemically synthesized colloidal quantum dots (CQDs) in a perovskite matrix has been considered one of the most promising approaches for the formation of IBSCs [1,4,5,7,19]. This approach benefits from the fact that both the halide perovskites and CQDs can be solution processed at low temperatures and allow easy combination of both dots and host materials via simple wet-processing/deposition methods [30,33]. From previous integration studies of CQDs and halide perovskites, PbS dots were revealed to be a great option to embed in MAPbX-type perovskites due to two main reasons: the adequately low bandgap of PbS (~0.4 eV) in the IR (infrared) and the high structural compatibility (low lattice mismatch) between dots and host materials [7]. Furthermore, PbS QDs are good light absorbers and demonstrate the highest optoelectronic properties for PV applications compared to other QD materials [37].

Ideally, for the IB formation, the introduction of the CQDs in the perovskite matrix should form a dense but monodispersed array of individual (non-aggregated) QDs within the perovskite material. This would allow for the wavefunctions of the QDs’ ground-state levels to overlap and thus establish the intended delocalized mini-band [5,20]. However, the investigations thus far have shown that it is quite difficult to reduce (or even control) the aggregation of the QDs. Thus resulting in disorganized arrays that present strong structural mismatches with the matrix, giving rise to an undesirably high density of defects that can act as recombination centers [1,38]. Nevertheless, if such control is reached, the integration of the two materials can even bring structural advantages, as it has been found that the QDs can act as nucleation sites to initiate perovskite crystal growth, improving the crystallinity and stability of the host matrix [7,32]. However, this mechanism is crucially dependent on the concentration of the PbS QD inclusion, and previous studies have shown that it may only be possible at reduced concentrations below 1 mg/mL [39].

Furthermore, the previously mentioned phase stability of the QDs can help increase the phase stability of the perovskite, hindering the undesirable phase transitions upon environmental exposure. Adding more to the possible advantages of the integration of these two materials, QDs can reduce the ion migration and defects occurring in the perovskite, consequently reducing the issue of hysteresis in the devices, which has been another main drawback of PSCs [32].

The present article shows unprecedented results in the development of perovskite absorber films embedded with PbS colloidal dots (CQD@PVK), intended for PV applications, following the guidelines of previous theoretical studies performed by the authors [5]. First, the PbS CQDs synthesis method is presented, followed by the ligand exchange process, which allows the subsequent introduction of the dots in perovskite hosts for the realization and analysis of the CQD@PVK films. The changes in the optoelectronic properties of the composite films with different QD sizes and concentrations introduced in the host are then assessed. Notably, this study has specifically focused on significantly higher concentrations of embedded dots compared to the concentrations typically tested so far [15,16,39,40]. This deliberate approach aims to enable robust QD-mediated effects in the optoelectronic response of the composite films, resulting in pronounced sub-bandgap absorption and photocurrent generation (measured via a photoconductor with band structure depicted in Figure 1b) at energies below the host perovskite bandgap.

Overall, the results allowed a better understanding of the physical mechanisms and fabrication conditions that enable the desired enhancement of the optical properties, such as sub-bandgap absorption and the electrical response via increased NIR photocurrent mediated by the QDs’ confined ground-state levels.

## 2. Materials and Methods

### 2.1. PbS QD Synthesis

The chemicals used in the quantum dot synthesis were lead oxide (PbO), oleic acid (OA), octadecene (ODE), hexamethyldisilathiane (TMS), octane, and acetone. These were all purchased from Sigma Aldrich and used with no further processing or purification.

PbS–OA-capped colloidal QDs were synthesized by the hot injection method. The lead precursor solution was prepared by adding 223 mg of PbO, 1.260 mL of OA, and 9 mL of ODE to a 25 mL three-necked round-bottom flask. The mixture was kept constantly stirring under a vacuum in a Schlenk line at 120 °C for about 1 h until the PbO was completely dissolved. Before the injection of the sulfur precursor solution, a reaction temperature of 70 to 120 °C was set and stabilized under N_2_ flow.

The sulfur precursor solution was prepared in a glovebox just before injection by mixing 2.5 mL of pre-degassed octadecene (1 h under constant stirring and vacuum at 120 °C) and 105 µL of TMS. The mixture was transferred to a syringe to inject into the lead precursor solution. Afterward, the sulfur precursor solution was swiftly injected into the lead precursor solution, and a sequence of color transitions from clear to yellow and from yellow to brown was noticed, indicating the formation of QDs in the mixture.

After injection, the heat source was immediately turned off, leaving the mixture to cool down slowly until a low temperature was reached (~50 °C). The mixture was further transferred to a 45 mL centrifuge tube, and 32.5 mL of acetone was added to precipitate the OA-capped dots. These QDs were centrifuged at 6000 rpm for 5 min. Afterward, the supernatant was discarded, and the precipitate was redissolved in 10 mL acetone, followed by 35 mL of acetone and further centrifugation at 6000 rpm for 5 min. The previous re-dissolution and precipitation step was repeated once more, but now the precipitate was dissolved into 2 to 3 mL of dry octane, filtered through a 220 nm PTFE filter, transferred to a glass flask, and stored in a freezer until used. Further characterization and optimization of the PbS QDs are presented in Section S1 of the Supporting Information.

### 2.2. Ligand Exchange

**Figure 2:**
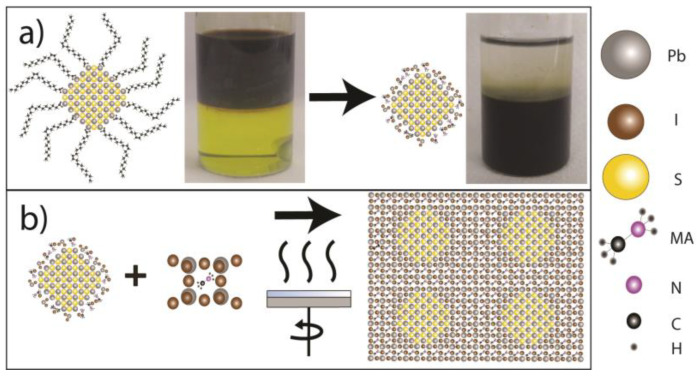
(**a**) Ligand exchange (LE) process showing pre-LE OA-coated PbS QDs in octane (left, top phase) and post-LE PbS QDs in DMF (right, bottom phase). (**b**) Schematic of the spin-coating and annealing process for PbS CQD@PVK film formation and a sketch of ideal QD insertion in MAPbI_3_ perovskite matrix.

The ligand exchange (LE) process (depicted in Figure 2a) used lead iodide (PbI_2_) from Sigma Aldrich and methylammonium iodide (MAI) from both Sigma Aldrich (Darmstadt, Germany) and Greatcell Solar (Queanbeyan, New South Wales, Australia). It also used N,N-Dimethylformamide (DMF), chlorobenzene and toluene from Thermo Scientific (Waltham, MA, USA).

Inside the glove box, 0.25 M of MAPbI_3_ was prepared by adding 115.25 mg of PbI_2_ and 39.75 mg of MAI to 1 mL of DMF and keeping it under strong stirring at 60 °C overnight. Afterward, 1 mL of 27.5 mg/mL of PbS QDS in octane (the apolar phase) was further added dropwise on top of the polar phase inside a 5 mL Eppendorf tube. It was then stirred overnight at room temperature. After a couple of hours, the top phase (lighter than DMF) had turned completely clear, while the bottom polar phase was completely brown, which indicates a successful ligand exchange of the QDs, as shown in Figure 2a. The top octane phase was discarded, and the bottom phase was washed twice by adding 1 mL of octane and vigorously shaking for 30 s, followed by discarding the top phase after phase segregation. The remaining perovskite-shelled QDs were precipitated by the addition of 0.6 mL of toluene and by a 10,000-rpm centrifugation for 5 min. Finally, the capped dots were dried in a vacuum and kept in powder form inside the glovebox.

### 2.3. Film Preparation

For the film preparation, spin coating and annealing were employed, as illustrated in Figure 2b. DMF was used as the solvent, while PbI_2_, MAI, and the previously synthesized PbS quantum dots were used as the materials.

Inside the glove box, 1 M of MAPbI_3_ in DMF was prepared by adding 461 mg of PbI_2_ and 159 mg of MAI to 1 mL DMF. This was followed by placing the solution under strong stirring overnight at 60 °C. Posteriorly, the solution was filtered through a 220 nm PTFE filter. For the preparation of the QDs in MAPbI3 films, 70 and 155 mg/mL of powdered shelled QDs were added to 1 M MAPbI_3_ solutions.

For film deposition, 110 µL of precursor ink (1 M of MAPbI_3_ in DMF with or without shelled QDs) was added to clean and UV–ozone-exposed soda-lime glasses (2.5 × 2.5 cm) and spun at 2000 rpm for 10 s. Then, the spin speed was increased to 4500 rpm, and 100 µL of antisolvent was dripped into the substrate and left to spin for 20 more seconds. After spinning, the films were annealed at 100 °C for 5 min. The resulting CQD@PVK film thicknesses are presented in Table 1. Films incorporated with higher CQD densities exhibit significant variability due to their considerable roughness.

### 2.4. Device Fabrication

The photodetectors were produced by e-beam evaporation of 100 nm of gold on top of the CQD@PVK or MAPbI_3_ (perovskite-only) films. This deposition process was carried out in a homemade evaporator using shadow masks, yielding 0.875 mm^2^ test devices contacted by interdigitated electrodes with ~125 µm of inter-finger distance and 7 mm in total length.

### 2.5. Characterization

The X-ray diffraction equipment used was PANalytical (Malvern, UK) Xpert PRO MRD, using a Cu K-alpha X-ray source.

The ultraviolet–visible–near-infrared spectrophotometry was performed using Perkin Elmer (Waltham, MA, USA) Lambda 950 equipment, equipped with an integrating sphere for the films’ characterization. For the PbS CQD characterization in solution, the dispersion was diluted in toluene to about 0.25 mg/mL of PbS/OA mass in solution.

The PL emission spectroscopy was performed in a SPEX^®^ Fluorolog^®^-3 FL3-22 HORIBA (Kyoto, Japan) spectrofluorimeter using 300 nm of excitation wavelength for visible range emission and 400 nm excitation wavelength for near-infrared range emission. For the solution characterization, a fixed concentration of 0.25 mg/mL of PbS/OA in toluene was used.

The film thicknesses were measured with a profilometer (Ambios XP Plus 200 Stylus (Santa Cruz, CA, USA).

The photodetector test devices produced as described in Section 2.4 were characterized by illumination with a 1-Sun spectrum close to AM 1.5 G, 740 nm, and 940 nm LED light sources using an Newport ORIEL Vera Sol (Irvine, CA USA) LSH-7520 LED-based Solar Simulator and were characterized electrically by an Agilent (Santa Clara, CA, USA) 4155C analyzer. The photocurrent measurements were performed by applying 2 V between the device terminals and measuring the current signal over time. The illumination was performed using chopped light at 0.680 Hz, starting at 940 nm, 740 nm, and then AM 1.5 G to avoid light degradation. The different illuminating spectra are presented in Section S2 of the Supporting Information.

Transmission Electron Microscopy was performed with a JEOL (Tokyo, Japan) JEM 2100 at 80–200 kV, analyzing both close-packed colloidal films of the as-synthesized PbS QDs drop-casted in the TEM grid as well as cross-sections of externally prepared lamellae (~100 nm thick) of CQD@PVK films.

## 3. Results and Discussion

### 3.1. Structural Properties of the Composite Films

For the PbS CQD@PVK films, two QD sizes (3 and 3.5 nm) were synthesized for inclusion in the perovskite matrix with two different concentrations: 70 mg/mL and 155 mg/mL.

**Figure 3:**
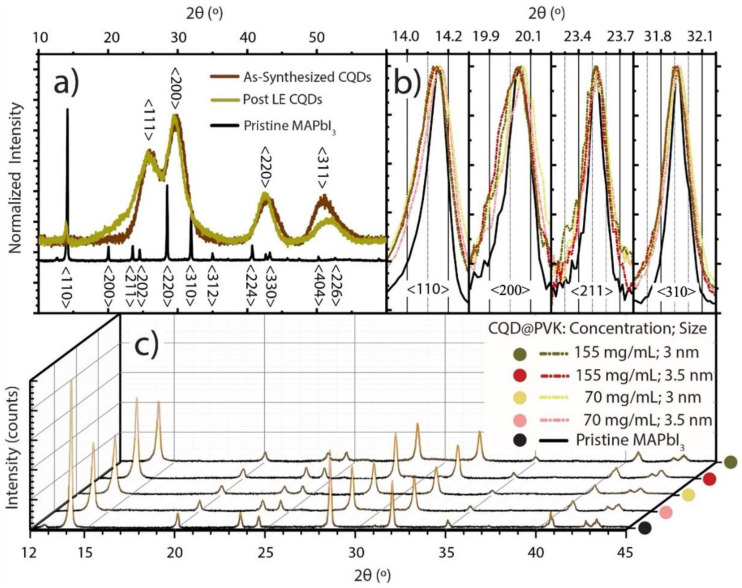
(**a**) Normalized XRD data of drop-casted OA-coated (as-synthesized) PbS CQD films and PbS CQD films after ligand exchange along with pristine MAPbI_3_. (**b**) Normalized XRD peaks of <110>, <200>, <211>, and <310> XRD peaks of typical MAPbI_3_ planes in pristine MAPbI_3_ (solid black line) versus those in the PbS CQD@PVK films (dashed colored lines). (**c**) Full XRD data of the same films with varying PbS CQD content and diameter.

The XRD analysis showed that the crystal structure of the OA-capped PbS CQDs presents diffraction peaks in the angles of 25.94°, 30.02°, 43.02°, and 50.86° (Figure 3a), which correspond to the <111>, <200>, <220>, and <311> planes of the cubic phase of PbS, respectively [43]. The cause for the broadness of these peaks lies in the fact that the as-synthesized drop-casted CQD films have a very small and randomly oriented crystallite size, along with a considerable amount of amorphous coordinating ligands on the surface (OA). After ligand exchange, the drop-cast films conserve the presence of the bulk PbS peaks together with a small degree of interference from the MAPbI_3_ diffraction pattern. This interference is caused by the presence of sufficient MAPbI_3_ material around the post-LE dots, resulting in small crystalline domains formed on the PbS surface during LE or drying of the drop-casted films [44]. As seen in Figure 3a, this results in the appearance of a small <110> peak at 14.15°, a higher bump at 20–24° likely caused by the <200> and <211> MAPbI_3_ peaks, a slight shift towards lower angles at 43.02° caused by the <224> MAPbI_3_ peak, and a shift towards higher angles at 50.86° potentially caused by <226> peak.

In the MAPbI_3_ perovskite films and CQD@PVK films, the existence of the tetragonal phase of MAPbI_3_ is confirmed through the sharp peaks at angles of 14.15°, 20.05°, 23.55°, and 31.95° for all samples, corresponding to the <110>, <200>, <211>, and <310> planes [44,45]. However, the peaks reveal a lower intensity (Figure 3c) and higher broadness (Figure 3b) in the CQD@PVK samples, indicating that the crystallization was perturbed by the CQDs’ embedment, leading to a higher bulk defect density. This is in accordance with previous CQD@PVK tests presented in the literature, from which it is expected that concentrations on the order of 1 mg/mL or higher (as used in this work) can significantly reduce the structural quality of the composite films [1,4,46]. Nevertheless, when comparing the patterns of the CQD@PVK films, there is an increase in peak intensity with higher dot concentration, particularly for the 3.5 nm QDs, which is due to a more intense signal caused by a higher film thickness (see Table 1). Additionally, it is worthwhile to note that PbS peaks are not present in the CQD@PVK films, which is attributed to the much smaller fraction of PbS in the films compared to MAPbI_3_ (~10% solid mass in solution for 70 mg/mL and ~20% for 155 mg/mL). This, together with the aforementioned low crystallite size and random distribution of PbS CQDs in the CQD@PVK films, resulted in relatively high broadness and low signal intensity in the XRD patterns.

### 3.2. Optical Assessment of CQD@PVK Films

**Figure 4:**
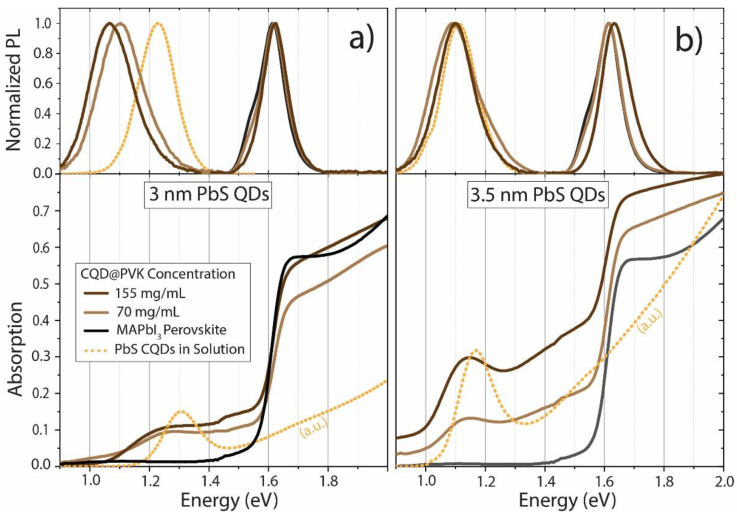
Normalized photoluminescence, PL (top panels), and absorption spectra (bottom panels) of the produced CQD@PVK films with 3 nm (**a**) and 3.5 nm (**b**) of PbS CQD diameters at different concentrations, as compared to the CQDs dispersed in toluene solution and to the pristine (PVK-only) MAPbI_3_ films.

The optical absorption and photoluminescence spectra (Visible–NIR) of the composite films are shown in Figure 4, compared to the spectra of the as-synthesized CQD solution in toluene. Notably, for both QD sizes and concentrations in this study, the absorption and emission properties of the constituent materials (PbS QDs and MAPbI_3_) can be observed and clearly distinguished in the CQD@PVK composites. Specifically, the CQD@PVK composites exhibit the band-edge absorption and emission of MAPbI_3_ at 1.60 eV (host bandgap), as well as the ground-state absorption of the PbS QDs at 1.23 eV and 1.18 eV for 3 nm and 3.5 nm dots, respectively. Additionally, there is a noticeable redshifted PL emission from the former.

There is an almost perfect spectral match of the absorption onset and PL peaks corresponding to the host bandgap transition (~1.6 eV) in the samples with and without CQDs, which reveals that the CQD incorporation generally preserves the main structural quality of the host perovskite. The only exception is the CQD@PVK film with a higher CQD size (3.5 nm) and higher concentration (155 mg/mL). Compared to the pristine MAPbI_3_ film, this particular film presents a slight blue shift (15 meV) in the perovskite PL peak position. This can be attributed to a higher degree of structural disorder in the CQD@PVK films when compared to pristine MAPbI_3_, as mentioned in the previous section.

A particularly relevant aspect is that the CQD@PVK films provide simultaneous emission peaks from the CQDs’ ground states (at ~1.1 eV) and from the surrounding perovskite host (at ~1.6 eV). This reveals that there is a significant energetic separation (represented by E_CI_ in Figure 1) with a small density of states between the energetically aligned ground states of the QDs (forming the IB) and the MAPbI_3_ conduction band (CB), which enables radiative emission from the CB to VB (host PL peak) and from the IB to VB (QDs PL peak).

In terms of the PL emission peaks of the PbS QDs, there is a notable redshift when these nanocrystals are included in the MAPbI_3_ structure. This could be due to the closer proximity of the dots when present in the solid film (as well as some degree of CQD agglomeration), thus increasing the probability of dot emission, absorption, and reemission at lower energy due to Stokes shift [47]. For further discussion on this matter, see Section S1 of the Supplementary Information. Another mechanism that can contribute to the emission and absorption redshift is the possible reduction in quantum confinement. This reduction may occur due to dot agglomeration and a decrease in the potential barrier to the surrounding environment. In their as-synthesized state, QDs are surrounded by electrically insulating OA, but upon inclusion in MAPbI_3_, this square-well-like barrier is lowered [48].

By comparing the emission of PbS QDs in toluene solution with that of CQD@PVK films, there is a general increase in redshift with higher QD spatial density from solution to films. This is especially true in the case of 3 nm QDs, where this effect is more prominent, which might be related to a higher surface-to-volume ratio for smaller dots and, thus, a higher dependence on the surrounding medium properties on their optical response [48].

In terms of absorption, when a higher CQD concentration is used, higher overall absorption is generally verified across the spectrum (below and above the PVK bandgap), as expected due to the high absorption coefficient of PbS. Nevertheless, other fabrication-related effects can influence these results. Namely, when incorporating higher dot concentrations, the solution with ligand-exchanged CQDs presented higher ink viscosity, which favored increased film thicknesses and roughness (see Table 1). The only exception occurred with the film with 70 mg/mL of 3 nm PbS QDs, which revealed relatively lower above-bandgap absorption. This is potentially caused by a reduction in the average crystallite size, as confirmed by the lower XRD peak intensities shown in Figure 3c for this sample.

Concerning the NIR absorption of the QDs in the composite films below the PVK bandgap, there is a proportional increase (>2-fold) of the ground-state peak intensity of the 3.5 nm dots with the higher (more than double) PbS mass content in the precursor ink. However, in the case of the 3 nm PbS QDs, the increase is smaller, which may be related to a higher degree of aggregation of the smaller QDs in the precursor ink. Such aggregation could lead to an ejection of the aggregates from the glass substrates during spin-coating and, thus, a lower mass concentration of PbS QDs in the formed film [32,33,34].

### 3.3. Photoconductivity of CQD@PVK Films

To assess the electrical response of the composite absorbers, simple photodetector device structures were produced by e-beam evaporation of ~100 nm of gold as contacts on top of the MAPbI_3_ and CQD@PVK films, as presented in Figure 1b. The characterization of the produced photodetectors was carried out by current measurements with a constant applied voltage of 2 V under different illumination conditions. Before illumination, the current under constant applied voltage was left to stabilize, as it decays over time when voltage is applied in the dark. This behavior is possibly a consequence of ionic migration, methylammonium reorientation within the MAPbI_3_ crystalline lattices, and charge accumulation in the polycrystalline film grain boundaries [49].

**Figure 5:**
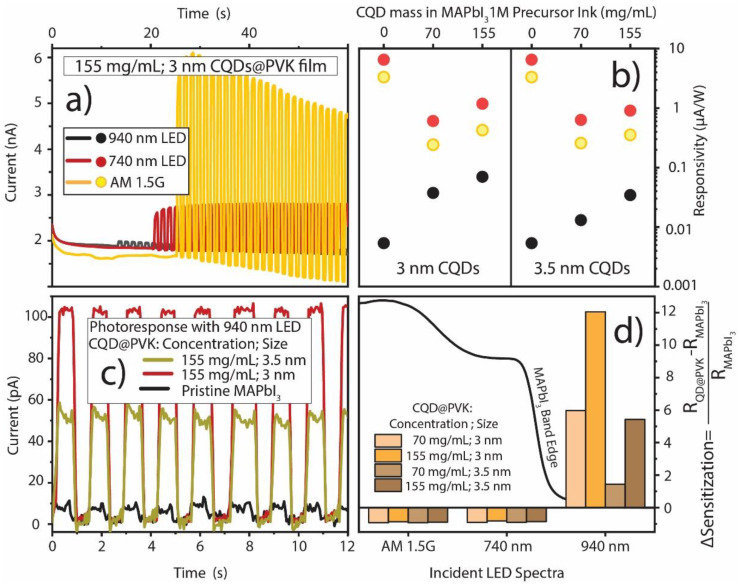
(**a**) Example of untreated current over time measurements for the produced CQD@PVK photodetectors with 3 nm PbS dots at 155 mg/mL concentration in the precursor ink under exposure to chopped LED light of different wavelengths, namely 940 nm (sub-bandgap) and 740 nm (at bandgap), as well as with the 1-Sun (near AM 1.5 G) spectra. (**b**) Responsivity (R) comparison between the photodetectors composed of the PbS QDs, with the different concentrations and diameters studied in this work, for the three incoming light spectra labeled in (**a**). (**c**) Treated NIR photoresponse of the CQD@PVK photodetectors at 155 mg/mL of PbS QD content, with two different QD diameters, versus pristine MAPbI_3_. (**d**) Sensitization (signal change enhancement due to CQDs) of the produced CQD@PVK films relative to the pristine MAPbI_3_ for the three different illumination conditions.

After stabilization, the photodetectors were illuminated by chopped light from three main sources: a 940 nm LED (with a 900 nm low-pass filter), a 740 nm LED, or the terrestrial 1-Sun (AM 1.5 G) spectrum from an LED-based solar simulator lamp. The current signal was acquired, as shown in the example of Figure 5a.

The evaluation of the overall signal increase (presented in Figure 5b) was performed by fitting the upper and lower envelope functions of the current obtained under chopped illumination, subtracting such functions, and then averaging the ON/OFF difference. This was then divided by the incident illumination power to obtain the responsivity (R, in A/W). Figure 5c shows examples of the NIR response after lower envelope function subtraction.

From the measurements performed, it is clear that there is a strong increase in sub-bandgap photoconductivity (observed for the 940 nm LED illumination) in films with QD inclusion, which can be attributed to sub-bandgap exciton generation and conduction band population, leading to increased photocurrent when the sample is irradiated. This is the signature of active IB-related mechanisms occurring in the nanostructured films.

Higher CQD concentrations in the films yielded a higher sub-bandgap photoresponse, as expected for a higher volumetric fraction of NIR absorbers (CQDs). In Figure 5d, it is possible to observe a particularly pronounced ΔSensitization in the NIR (940 nm LED) for the 3 nm CQDs. Although in Figure 4 it is possible to observe lower QD absorption in the NIR for these dots, this more pronounced electrical result may be due to the better alignment of the incoming photon energy (at 1.32 eV) with the ground-state peak of the 3 nm QDs in the film (E_0_~1.26 eV). At the same time, a larger misalignment occurs for the 3.5 nm QDs in film (E_0_~1.14 eV). Better energetic alignment potentiates more direct (radiative) VB to IB transitions in the semiconductor instead of exciting carriers to other energetic states between bands, followed by further excitation from the IB to the CB, which is likely thermally assisted [4]. With more transient processes and the lifetime of an excited carrier in the semiconductor, the lower the probability of recombination and, thus, the higher the photoresponse of the film.

In terms of above-bandgap photoresponse (740 nm LED and AM 1.5 G), no significant differences are seen between the two diameters of CQD@PVK films, as the main source responsible for photogeneration in the visible range is the surrounding perovskite host. However, an increase in the visible response is observed with a higher concentration of QDs, which is mainly attributed to the higher thickness (Table 1) of CQD@PVK films, as mentioned in the optical analysis of Figure 4. For all the samples, it is noteworthy that the higher responsivity is achieved for the 740 nm incoming light due to the close proximity of such an LED illumination peak to the MAPbI_3_ bandgap (see Figure S3 in Supplementary Information), thus providing a favorable amount of carrier generation slightly above the conduction band minimum. On the other hand, with the broadband AM 1.5 G irradiation, the incoming photons are much more spectrally dispersed towards energies at which the material presents a low absorption coefficient (e.g., below bandgap). Consequently, the measured current over incident power (responsivity) is reduced.

Although sub-bandgap light seems to cause an increase in photoconductivity in films with QD inclusion versus pristine MAPbI_3_, there is also an evident decrease in photoconductivity for the 740 LED and AM 1.5 G spectrum. This is suspected to be a consequence of the disruption of the crystalline structure of MAPbI_3_ when relatively high amounts of QDs are included in the films. This high degree of QD embedment causes the formation of smaller crystalline domains, increasing the number of grain boundaries and a higher amount of QD aggregates. The latter is also a cause of deconfinement, as the QDs in aggregates can lose their quantum-well-like electronic structure and start behaving more similarly to highly defective bulk PbS, acting as recombination centers for excitons in the CQD@PVK absorbers [50].

It is also important to note that, even for sub-bandgap energies, the absorption coefficient of MAPbI_3_ is always non-zero. This results in a measurable amount of sub-bandgap light absorption mainly attributable to the polycrystalline nature of the perovskite films, originating tail effects in the absorption spectrum caused by in-gap defect/interface states present in the bulk material [51]. Consequently, a very slight photoresponse is observed below the MAPbI_3_ bandgap in pristine perovskite films, even without QDs [52].

## 4. Conclusions

The exploration of quantum effects with nanostructured absorber films is emerging as a new hot topic in optoelectronic-related fields, such as photovoltaic cells, with better capabilities for exploiting the spectrally broad sunlight range. For that, solution-processed materials, such as perovskite hosts embedded with colloidal PbS QDs, appear to be a promising combination for the realization of semiconductor absorbers, enabling photo-carrier generation from light with energy below their bandgap. Previous attempts in the literature have tested these types of heterogeneous materials for different optoelectronic applications. However, there is a strong potential, particularly for the realization of the long-sought intermediate band solar cell concept, as explored here.

In this regard, the present study presents an effective methodology to produce CQD@PVK composite films with a high density of embedded CQDs, as required to reinforce the photoresponse resulting from the carrier transitions enabled by the quantum-confined CQD levels. Such response is analyzed for different main physical parameters of the composite material, namely different CQD sizes and dots’ concentrations in the host, seeking not only the best incorporation methods but also to better understand the effects of these parameters on the films’ optoelectronic properties.

Although the inclusion of PbS QDs in a MAPbI_3_ matrix was overall successful, there is still much room for process improvement as CQD aggregates are present and the perovskite crystallinity is disrupted. Nevertheless, when optically probing the fabricated composite films, the conservation of the absorption onset and the PL emission peaks of both MAPbI_3_ and PbS QDs can be clearly verified.

In terms of electric response, photocurrent measurements performed in CQD@PVK photodetectors revealed strong sub-bandgap photogeneration (namely for incident NIR light at 1.32 eV, 940 nm wavelengths) compared to PVK-only devices, which points towards the desired IB-mediated absorption process. This process is enabled by the establishment of an in-gap energy level caused by the energetically aligned CQD ground states in the composite materials. These findings are similar to the effects observed in previous studies [1] on CQD@PVK materials.

To conclude, this study shows the feasibility of realizing CQD@PVK absorbers with a high concentration of embedded dots, which allows amplifying the optoelectronic effects caused by the dots’ carrier confinement properties. Although the fabrication method still needs development to realize this novel technology to its full potential, the results here presented contribute to a better understanding of the physical mechanisms at play in nanostructured dots-in-host films while opening important avenues for further exploration in future works.

## Figures and Tables

**Table 1:** Measured average and maximum deviation of the deposited films’ thicknesses.

Sample (Dots’ Concentration, Size)	Film Thickness (nm)
Pristine MAPbI_3_ Perovskite (PVK)	249 ± 7
CQD@PVK (70 mg/mL, 3 nm dots)	399 ± 27
CQD@PVK (70 mg/mL, 3.5 nm dots)	394 ± 27
CQD@PVK (155 mg/mL, 3 nm dots)	419 ± 32
CQD@PVK (155 mg/mL, 3.5 nm dots)	594 ± 100

## Data Availability

Data sharing is not applicable to this article.

## Supplementary Materials

The following supporting information can be downloaded at: https://www.mdpi.com/article/10.3390/nano13172447/s1, Figure S1: Optical Characterization of chemically synthesized PbS CQDs; Figure S2: Transmission Electron Microscopy of dropcasted PbS CQDs and cross section of a CQD@PVK film; Figure S3: Incoming Light Spectra used to characterize IB Photodetectors.
