# Supplementary material for: Sub-Bandgap Sensitization of Perovskite Semiconductors via Colloidal Quantum Dots Incorporation
Source: Nanomaterials (Basel). 2023 Aug 29;13(17):2447. doi: 10.3390/nano13172447 (PMC10489900; doi:10.3390/nano13172447)
Supplement: Supplementary file 1 [file nanomaterials-13-02447-s001.zip › nanomaterials-2550703-supplementary.pdf]

Supplementary Information

Sub-Bandgap Sensitization of Perovskite Semiconductors via Colloidal Quantum Dots Incorporation

G. Ribeiro [1,2,,†], G. Ferreira [1,†], U. D. Menda [1], M. Alexandre [1], M. J. Brites [3], M. A. Barreiros [3], S. Jana [1], H. Águas [1], R. Martins [1], P. A. Fernandes [2,4,5], P. Salomé [2,6] and M. J. Mendes [1,*]*

[1] i3N/CENIMAT, Department of Materials Science, NOVA School of Science and Technology and CEMOP/UNINOVA, Campus de Caparica, 2829-516 Caparica, Portugal
[2] INL, International Iberian Nanotechnology Laboratory, 4715-330 Braga, Portugal
[3] LNEG, Estrada do Paço do Lumiar, 22, 1649-038 Lisboa, Portugal
[4] CIETI, Departamento de Física, Instituto Superior de Engenharia do Porto, Instituto Politécnico do Porto, 4249-015 Porto, Portugal
[5] Departamento de Física, Universidade de Aveiro, Campus Universitário de Santiago, 3810-193 Aveiro, Portugal
[6] i3N, Universidade de Aveiro, Campus Universitário de Santiago, 3810-193 Aveiro, Portugal
* Correspondence: ga.ribeiro@campus.fct.unl.pt (G.R.); mj.mendes@fct.unl.pt (M.J.M.)
† These authors contributed equally to this work.

S1. Quantum dots synthesis

A main parameter studied in this work was the size of the PbS QDs dispersed in the $MAPbI_3$ matrix. As mentioned earlier, this size is directly related to the light absorption profile of the semiconductor heterocrystal composites. Since the colloidal QD dispersions used in this study were produced in-house, a reliable synthesis and an understanding of their physical properties is essential as the starting point of the present experimental work.

QDs of different sizes were obtained through synthesis at different temperatures. For the purpose of estimating the average QD size in each dispersion, NIR-Visible absorption spectroscopy is used to first determine the value of E_0 (first exciton) from the spectral position of the first maxima at lower energies (e.g. E_0~1.15 eV in Figure S1 b). The resulting values of E_0 are expected to be directly related to the average QD diameter (*d*) in each dispersion, according to following empirical expression [1]:

$$E_0 = 0.41 + \frac{1}{0.0252d^2 + 0.283d}$$

From this empirical expression the value of *d* is estimated in each QD solution.

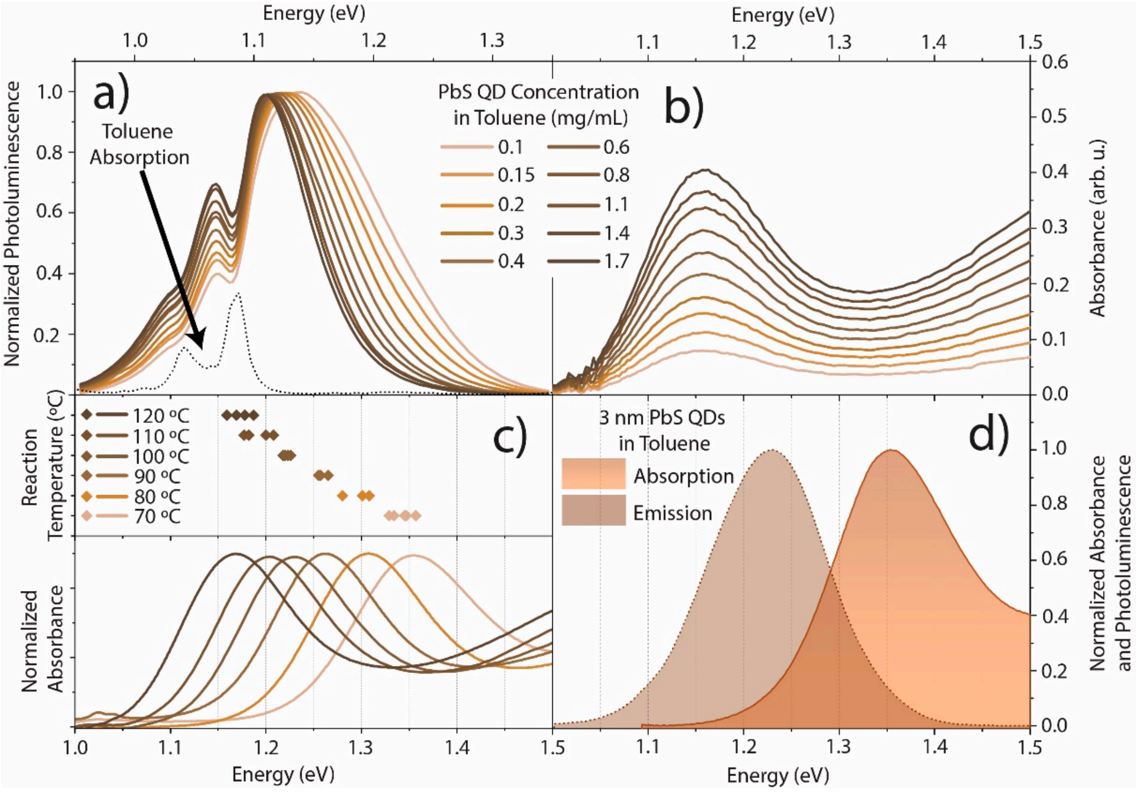

Figure S1. Normalized PL **(a)** and Absorption **(b)** of different concentrations of PbS QDs in Toluene. Spectral position of the ground-state absorption peaks of PbS QDs synthesized at different temperatures **(c)**. Measured Absorption and Emission for the example of CQDs with 3 nm diameter, showing a Stokes Shift of 125 meV **(d)**.

For the size distribution optimization, the only varied parameter was the injection temperature - higher injection temperatures yielding higher diameter of the colloidal PbS QDs, meaning lower E_0 but retaining the distribution broadness for the studied range of QDs as seen in Figure S1 c).

In a similar way as with the diameter study, the optical behaviour of the QDs in different concentrations was investigated through NIR Photoluminescence and Optical Absorption techniques, as shown in Figure S1 a) and b), respectively. From the absorbance of increasing concentrations of PbS QDs in solution, it is notable that only the absorption intensity increases with higher concentration, while the peak position of E_0 energy remains unchanged, evidencing sufficient passivation of the PbS QDs surface by the peripheral OA, for the studied concentration of PbS QDs in solution.

Comparing absorption to photoluminescence emission spectra, it is possible to verify the red-shift of the PL ground-state peak relative to the corresponding absorption peak (E_0), shown in Figure S1 d). This Stokes Shift is commonly present in semiconductor nanocrystals and other materials, being typically more pronounced for smaller particles owing to their higher surface to volume ratio [2].

It is also notable the concentration dependence of the central emission energy of PbS QDs in solution, which occurs due to the inherent variability in diameters and shape of the produced QDs, and the overlap of the absorption and emission spectra of a given population of QDs in solution (intersecting area in Figure S1 d), i.e. higher E_0 QDs emit photons that can be reabsorbed by lower E_0 QDs (bigger diameter). The absorbed photons can cause a re-emission of a recycled photon with energy equal to E_0 of the given QD minus its Stokes Shift. This process repeats until the recycled photon cannot cause excitation of a QD in a given population. By having a lower concentration of QDs in solution, the

probability of photon recycling is lowered, thus having a more blue-centered emission and a more accurate value of Stokes Shift can be estimated.

S2. TEM Analysis

TEM image analysis conducted on the as-synthesized (Figure S2 a)) PbS CQDs, drop-casted in a TEM grid, reveals a good distribution of individual CQDs with mono-dispersed sizes arranged in an hexagonal close-packed array. The zoomed image (Figure S2 b)) shows finer details where the different colourations correspond to different crystalline orientations of the CQDs.

Cross-section TEM was also performed on prepared lamellae of the CQD@PVK films. The similarity of the Pb-Pb distances in the dots and host materials, as well as random QD nanocrystallite orientation, make it challenging to distinguish individual embedded CQDs. Noticeably, darker regions of PbS QDs are visible in Figure S2 c), which appear to be clustered since the size of these structures is bigger than a 3.5 nm QD. However, the observed TEM cross-section is a projection from beam transmission through the ~100 nm thick lamellae, which can result in apparent visualization of CQD clustering for isolated dots along the lamellae depth, particularly with high QD densities in the films (as in the present case). Indeed, for the bigger 3.5 nm QDs analysed by TEM in Figure S2, the optical properties of the CQD@PVK films (see Figure 4) reveal only marginal shifting of the absorption and emission peaks associated to the CQDs' ground-states, relative to the nanoparticles in solution. Therefore, such optical response indicates that the dots are mostly well dispersed throughout the composite films.

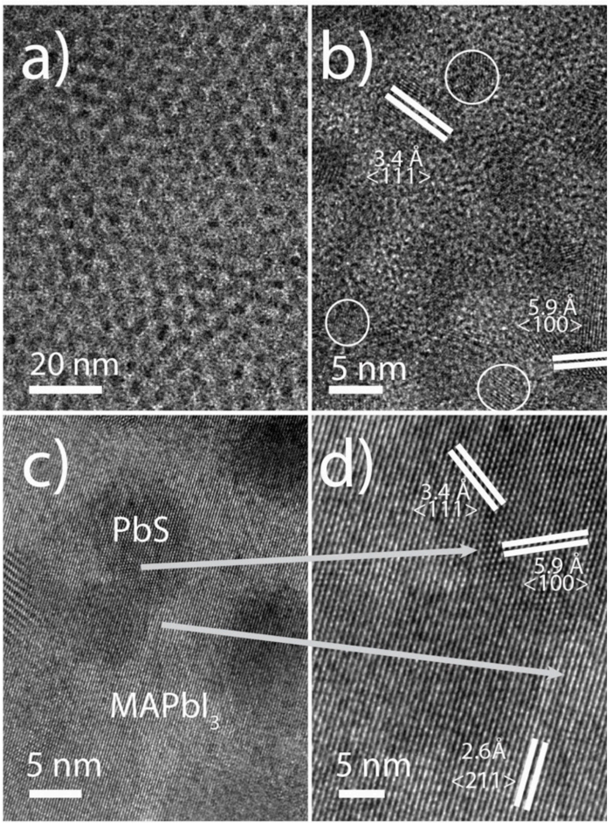

Figure S2. TEM images at different magnification of 3.5 nm PbS CQDs, as-synthesized (a,b) and dispersed in a $MAPbI_3$ matrix (c,d).

S3. Incident Light Spectra for Photoconductivity

In Figure S2 the light spectra of the used LED sources are shown, denoting the non-intersection of the NIR (940 nm wavelength) LED with the $MAPbI_3$ bandgap (1.60 eV, 775 nm wavelength) which was also ensured by the use of a cut-off filter for wavelengths shorter than 900 nm.

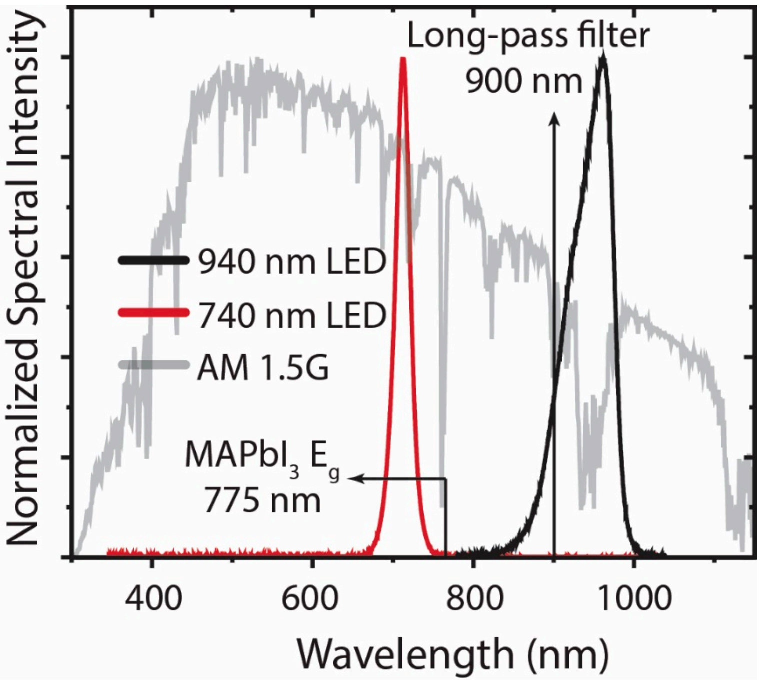

Figure S3. Spectra of light sources used for the photodetector measurement, from a LED-based Solar Simulator.

References

[1] I. Moreels *et al.*, “Size-Dependent Optical Properties of Colloidal PbS Quantum Dots,” *ACS Nano*, vol. 3, no. 10, pp. 3023–3030, Oct. 2009, doi: 10.1021/nn900863a.

[2] F. Xu, L. Gerlein, X. Ma, C. Haughn, M. Doty, and S. Cloutier, “Impact of Different Surface Ligands on the Optical Properties of PbS Quantum Dot Solids,” *Materials*, vol. 8, no. 4, pp. 1858–1870, Apr. 2015, doi: 10.3390/ma8041858.
